# Assessment of Phosphate and Osmolarity Levels in Chronically Administered Eye Drops

**DOI:** 10.4274/tjo.galenos.2018.43827

**Published:** 2019-06-27

**Authors:** Onur Özalp, Eray Atalay, İbrahim Özkan Alataş, Zeynep Küskü Kiraz, Nilgün Yıldırım

**Affiliations:** 1 Eskişehir Osmangazi University Faculty of Medicine, Department of Ophthalmology, Eskişehir, Turkey; 2 Eskişehir Osmangazi University Faculty of Medicine, Department of Medical Biochemistry, Eskişehir, Turkey

**Keywords:** Phosphate, eye drops, osmolarity, corneal calcification

## Abstract

**Objectives:**

To assess phosphate and osmolarity levels of chronically administered eye drops commercially available in Turkey.

**Materials and Methods:**

A total of 53 topical eye drops including 18 antiglaucoma drugs, 4 nonsteroidal anti-inflammatory drugs (NSAIDs), 10 corticosteroids, 7 antihistaminics, and 14 artificial tears identified using the Vademecum Modern Medications Guideline (2018) were included in the study. Phosphate levels were assessed using Roche Cobas C501 analyzer (Roche Diagnostics GmbH, Mannheim, Germany) and the respective kits. Osmolarity was assessed using Vescor Vapro 5600 vapor pressure osmometer (Sanova Medical Systems, Vienna, Austria). Mean phosphate and osmolarity levels were obtained after averaging three measurements. Eye drops were categorized as isoosmolar, hypoosmolar and hyperosmolar based on physiologic tear osmolarity range (296.5±9.8 mOsm/L).

**Results:**

The highest phosphate concentration was found in the antiglaucoma group (20.3±35.4 mmol/L), followed by antihistaminics (17.3±17.9 mmol/L), corticosteroids (15.2±19.1 mmol/L), artificial tears (0.8±1.0), and NSAIDs (0.04±0.08). Percentage of medications in the hyperosmolar category was highest in the NSAI group (75%), followed by antihistaminics (43%), corticosteroids (20%), and antiglaucoma drugs (19%). Nearly all of the artificial tear formulations were in the hypoosmolar (71%) or isoosmolar (21%) categories.

**Conclusion:**

Approximately 40% of glaucoma medications and approximately 60% of corticosteroid and antihistaminic medications had a phosphate concentration higher than the physiologic tear phosphate level (1.45 mmol/L).

## Introduction

While topical eye drops have an important place in the treatment of eye diseases, long-term and inappropriate use may cause serious complications and side effects affecting the ocular surface. These side effects may be caused by an active pharmaceutical ingredient, preservative, or vehicle in the topical formulation.^1^ The side effect profiles of active ingredients are thoroughly investigated during the stages of drug development, and the process of monitoring for adverse effects also continues after the molecule enters the market. After recent studies revealed that preservatives can also cause severe toxicity, efforts have been made to develop less toxic preservative molecules or preservative-free eye drops. However, the potential toxicity of molecules comprising eye drop vehicles has been a relatively neglected topic that has not been given due importance.

Vehicles are involved in buffering eye drops and ensure that the formulation has the appropriate tonicity and viscosity.^#*#ref2#^*# Buffering agents include molecules like acetic, boric, and hydrochloric acid, potassium or sodium bicarbonate, phosphate, and citrate.^1^ Phosphate, a commonly used buffer, is a vehicle with high buffering capacity that stabilizes the pH level at 7.4, and can also be found in some formulations as part of the active ingredient.^1,3,4^ In addition, it has the added advantage of making corticosteroid-containing solutions more transparent.^3^

Although phosphate is an effective buffer, it interacts with calcium cations on the ocular surface to disrupt the structure of the precorneal tear film and form insoluble hydroxyapatite [Ca_5_(PO_4_)_3_OH] or calcium phosphate crystals in the cornea.^2,5,6,7^ The resulting crystals cause irreversible stromal opacification and reduced vision, and can also have a serious impact on patient comfort.^5,6,8^ An example of this crystallization was previously demonstrated in a patient with chemical burn of the ocular surface that was irrigated with a phosphate-buffered saline solution.^9^ The development of irreversible corneal calcification after the use of phosphate-buffered artificial tears for ocular surface disorders occurs for a similar reason.^5^ The extent of accumulation depends on factors such as the size of the epithelial defect, the presence of dry eye, the pH and tonicity of the formulation, and the frequency and duration of use.^2,9,10^

The aim of our study was to examine the phosphate concentrations and osmolarity levels of chronically administered eye drops commercially available in Turkey. We hereby intend to highlight the distinct importance of phosphate levels in eye drops in addition to the known hazards imposed by the active ingredients and preservatives.

## Materials and Methods

The Vademecum Modern Drug Directory (2018) was screened for antiglaucoma drugs, nonsteroidal anti-inflammatory drugs (NSAIDs), corticosteroids, antihistamines, and artificial tears for chronic topical use that are commercially available in Turkey. A total of 53 topical drugs, including 18 antiglaucoma drugs, 4 NSAIDs, 10 corticosteroids, 7 antihistamines, and 14 artificial tears, were included in the study in order to examine their phosphate and osmolarity levels (Table 1). Because this study did not involve humans or the use of human biological material, it was considered exempt from ethics board approval by the Ethics Committee of Eskişehir Osmangazi University. Topical formulations with high viscosity were excluded from the research due to technical reasons. Phosphate levels were determined at the Medical Biochemistry Department Laboratory of the Medical Faculty at Eskişehir Osmangazi University using a Roche Cobas C501 analyzer (Roche Diagnostics GmbH, Mannheim, Germany) with an inorganic phosphate kit based on the molybdate UV method.^11^ The kit has a measurement range of 0.1-6.46 mmol/L and a lower limit of detection of 0.1 mmol/L. Samples above the measurable range were diluted and analyzed again. The kit has good reproducibility (CV<1.5%). Osmolarity of the topical drops was evaluated with a Vescor Vapro 5600 model steam pressure osmometer (Sanova Medical Systems, Vienna, Austria) found in the same laboratory. Three levels of control were used to calibrate the device: low (100±2 mOsm/L), normal (290±3 mOsm/L), and high (1000±5 mOsm/L). The phosphate and osmolarity levels of each eye drop were determined three times and the average values were included in the analysis.

Information about the preservatives found in the drops was obtained from the Vademecum Modern Drug Directory.

Drops within the physiological osmolarity range of tears (296.5±9.8 mOsm/L)^12^ were classified as isoosmolar, and those below and above this range were classified as hypoosmolar and hyperosmolar, respectively.

Based on their phosphate concentrations, the topical drops were classified as being within physiological range (≤1.45 mmol/L), slightly high (1.45-25 mmol/L), moderately high (25-50 mmol/L), and very high (≥50 mmol/L).^2^

### Statistical Analyses

All statistical analyses were made with SPSS version 21.0 (SPSS, Inc. IBM, Chicago, IL). The average phosphate values of drugs with different preservative ingredients and in the different osmolarity categories were evaluated with Kruskal-Wallis test. Statistical significance was set at p<0.05.

## Results

**Figure 1:**
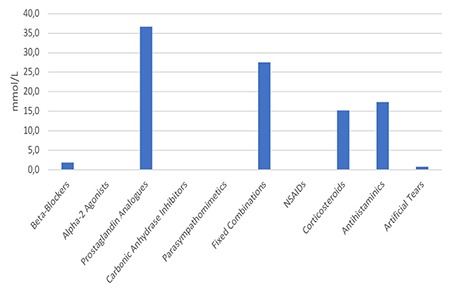
Mean phosphate levels of different drug groups NSAIDs: Nonsteroidal anti-inflammatory drugs

**Figure 2:**
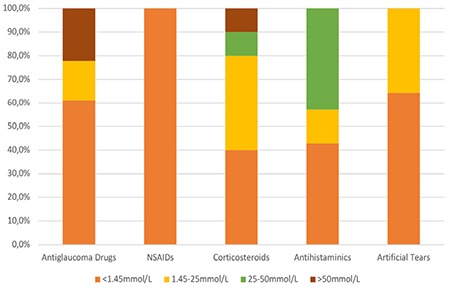
Percent distribution of phosphate levels in the different drug groups NSAIDs: Nonsteroidal anti-inflammatory drugs

The phosphate concentrations and osmolarity categories of the eye drops included in the study are summarized in Table 1. The highest measured average phosphate level was in the antiglaucoma group (20.3±35.4 mmol/L), followed by antihistamines (17.3±17.9 mmol/L), corticosteroids (15.2±19.1 mmol/L), artificial tears (0.8±1.0 mmol/L), and NSAIDs (0.04±0.08 mmol/L) (Figure 1). Thirty-one (58.5%) of the 53 topical drops contained phosphate levels within the physiological range. Preparations containing moderately and very high levels of phosphate accounted for 22.2% of the antiglaucoma drops and 42.9% of the antihistamines (Figure 2).

In the antiglaucoma group, it was noted that drops containing latanoprost contained especially high phosphate levels (Table 1). In the antihistamine group, drops containing olopatadine were found to contain high levels of phosphate, while other drops contained trace amounts of phosphate (Table 1). In the artificial tear group, most preparations had trace amounts of phosphate, while those containing sodium hyaluronate had slightly high levels of phosphate (Table 1).

**Figure 3:**
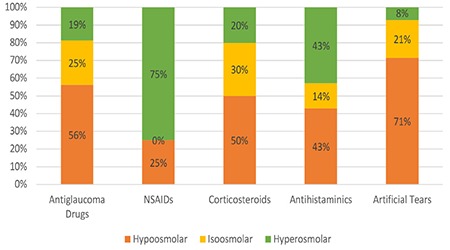
Percent distribution of osmolarity levels in the different drug groups NSAIDs: Nonsteroidal anti-inflammatory drugs

When different drug groups were evaluated based on their osmolarity levels, it was found that preparations in the NSAID group were of hyperosmolar character, while preparations in the artificial tear group were mostly hypoosmolar or isoosmolar (Figure 3).

**Figure 4:**
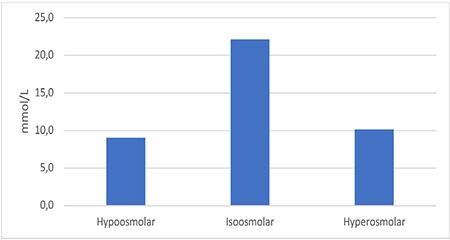
Average phosphate levels of drugs by osmolarity category

Evaluation of the phosphate levels of drugs in different osmolarity categories showed that hypoosmolar and hyperosmolar drugs contained similar levels of phosphate (9.0±24.6 mmol/L and 10.2±19.6 mmol/L, respectively); isoosmolar drugs had a relatively higher mean phosphate level (22.1±25.6 mmol/L), but the difference was not statistically significant (p>0.05) (Figure 4).

**Figure 5:**
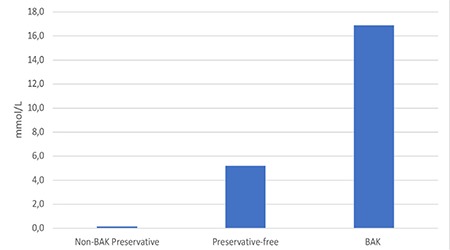
Average phosphate levels according to preservative BAK: Benzalkonium chloride

It was noted that of the 53 drugs, 34 contained benzalkonium chloride (BAK) as a preservative, 9 contained a non-BAK preservative, and the remaining 10 drugs contained no preservatives. When phosphate levels were evaluated based on preservative, the highest phosphate level was in those containing BAK (16.9±27.4 mmol/L), followed by preservative-free drops (5.2±14.4 mmol/L) and drops containing non-BAK preservatives (0.14±0.42 mmol/L) (Figure 5).

There was a significant difference between the group containing BAK and the group containing non-BAK preservatives (p=0.04), but the other comparisons did not yield statistically significant results.

## Discussion

Corneal calcification can result in severely reduced vision and in most cases irreversible corneal opacification, and may be associated with long-term use of eye drops with high phosphate content.^5,6,13^ The formation of crystals without visible calcification due to the use of high-viscosity artificial tears can also lead to irritation and thereby disrupt patient comfort.^8^ Rapid corneal calcification has also been reported in patients with large epithelial defects that were irrigated with phosphate-buffered solutions after chemical burns.^9,14,15^ In this study, we found that 22 (41.5%) of the 53 drops analyzed contained levels of phosphate exceeding physiological concentration (0-1.34 mmol/L) and that the majority of these were in the antiglaucoma and antihistamine drug groups.

The deposition of hydroxyapatite [Ca_5_(PO_4_)_3_OH] crystals in Bowman’s layer and the superficial stroma of the cornea is called band keratopathy.^16^ In cases with both epithelial damage and disruption of Bowman’s membrane, accumulation occurs in the deeper corneal stroma and Descemet’s membrane in the form of calcareous degeneration.^17^ The solubility of these crystals decreases with alkaline pH and high temperature.^18^ The pH value (physiological range 7.6-7.8)^19^ and tonicity of the ocular surface, extent of epithelial damage, and presence of inflammation and barrier dysfunction are among the factors responsible for the development of corneal calcification.^2,9^ Especially in dry eye patients, the tear film becomes more alkaline and hyperosmolar.^20,21,22^ An alkaline shift in the tear film has also been reported in association with age, independent of dry eye disease.^23,24^ The hyperosmolar state that occurs in dry eye disease is known to trigger the release of inflammatory mediators and proteases, which cause epithelial destruction.^25^ Similarly, topical drops with a hyperosmolar character have also been shown to alter tear osmolarity and increase inflammation.^26^ As ocular inflammation is a known risk factor for corneal calcification, hyperosmolar drops are not recommended for patients with a predisposition to corneal calcification.^27,28^

Although the phosphate content of the artificial tears analyzed in our study were within physiological limits, factors such as high-frequency use, inadequate lacrimal drainage, extended tear turnover time, and the high viscosity of artificial tears can increase the duration of contact between the ocular surface and the phosphate found in the formulation and thus lead to a tendency for calcification.^2,10^ Because dry eye also involves an inflammatory component, treatment may involve the intermittent use of steroids. Full-thickness calcification in the corneal stroma following the long-term use of dexamethasone phosphate was reported in a patient with Stevens-Johnson syndrome (SJS).^6^ Therefore, in the presence of an epithelial defect, it may be beneficial to prefer topical steroids that have low phosphate content or contain a non-phosphate buffer and are preferably acidic. Notably, the only BAK-free formulation in the topical steroid group contains phosphate at a concentration above the physiological limit.

In our study, the highest phosphate concentrations were detected in the antiglaucoma drops. Drops containing latanoprost in particular contained approximately 50 times more phosphate than the upper limit of the physiological range. Although the more acidic pH values (≈6.4)^10^ of these drops may seem like an advantage, the high phosphate concentrations increase their risks. In contrast, although bimatoprost drops and bimatoprost fixed combination drops contain less phosphate, they are more alkaline.^10^ The disclaimers in the package inserts of both of these prostaglandin analogues stating that “in rare cases, patients with severe damage to the cornea may develop cloudy patches due to the calcium build-up during treatment” should be evaluated in this context.^29,30^ Considering that predisposition to phosphate deposition in the cornea is a pH-dependent process, it will be valuable to demonstrate the effect of both preparations *in vivo*. In addition to drug pH values, the presence of ocular surface inflammation in glaucoma patients may be a factor that increases the risk of corneal deposits. Although trace amounts of phosphate were detected in the drops containing timolol in our study, accumulation in the superficial corneal stroma associated with timolol has been reported in the literature.^31,32^

Combining the reduced tear film breakup time, ocular pH changes, and ocular surface temperature and chronic inflammation that occur in allergic conjunctivitis with the chronic use of drugs containing high phosphate levels may promote the formation of corneal deposits.^33,34,35^ It is important to evaluate the topical antihistamines, steroids, and artificial tears used in treatment with this in mind. Shield ulcers that may occur in vernal conjunctivitis are another condition in which the risk of corneal disposition should be assessed.

## Conclusion

In summary, cases of acute or chronic corneal calcification associated with the use of topical drops or irrigation solutions with high phosphate levels have been reported in patients with chemical burns, dry eye, and chronic keratoconjunctivitis secondary to SJS.^5,6,9^ Considering evidence that phosphate-buffered tears but not citrate-buffered tears caused corneal calcification in some rabbits with mechanical abrasion-induced epithelial defect, drops containing a non-phosphate buffer can be considered for at-risk patients.^18^ A European Medicines Agency report evaluating 117 cases related to this topic emphasized that there is a possible association between corneal calcification and the use of topical drops in patients with corneal surface disorders.^36^ The reported concluded by stating that due to the very low risk, there is no need to refrain from using phosphate buffered drops, but that the risk-benefit balance should be considered when prescribing these drugs to patients with corneal damage.^36^ Knowing the chemical structure of topical formulations and selecting drops that have suitable tonicity and pH according to the disease profile and contain a buffer that will not promote accumulation will help prevent ocular surface complications associated with the use of eye drops.

## Figures and Tables

**Table 1:**
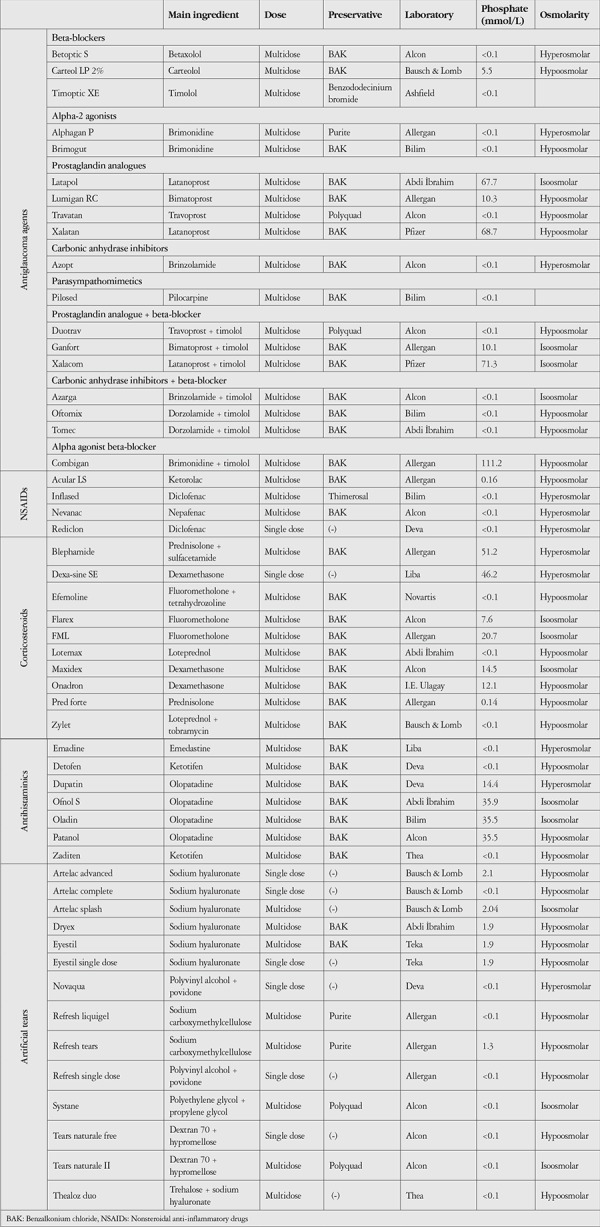
Phosphate and osmolarity levels in the different categories of eye drops
